# Cross-platform comparisons for targeted bisulfite sequencing of MGISEQ-2000 and NovaSeq6000

**DOI:** 10.1186/s13148-023-01543-4

**Published:** 2023-08-14

**Authors:** Jin Sun, Mingyang Su, Jianhua Ma, Minjie Xu, Chengcheng Ma, Wei Li, Rui Liu, Qiye He, Zhixi Su

**Affiliations:** Singlera Genomics (Shanghai) Ltd., No. 500, Furonghua Road, Shanghai, 201203 China

**Keywords:** Targeted bisulfite sequencing, cfDNA, MGISEQ-2000, Methylation, ctDNA, NGS, PDAC

## Abstract

**Background:**

An accurate and reproducible next-generation sequencing platform is essential to identify malignancy-related abnormal DNA methylation changes and translate them into clinical applications including cancer detection, prognosis, and surveillance. However, high-quality DNA methylation sequencing has been challenging because poor sequence diversity of the bisulfite-converted libraries severely impairs sequencing quality and yield. In this study, we tested MGISEQ-2000 Sequencer’s capability of DNA methylation sequencing with a published non-invasive pancreatic cancer detection assay, using NovaSeq6000 as the benchmark.

**Results:**

We sequenced a series of synthetic cell-free DNA (cfDNA) samples with different tumor fractions and found MGISEQ-2000 yielded data with similar quality as NovaSeq6000. The methylation levels measured by MGISEQ-2000 demonstrated high consistency with NovaSeq6000. Moreover, MGISEQ-2000 showed a comparable analytic sensitivity with NovaSeq6000, suggesting its potential for clinical detection. As to evaluate the clinical performance of MGISEQ-2000, we sequenced 24 clinical samples and predicted the pathology of the samples with a clinical diagnosis model, PDACatch classifier. The clinical model performance of MGISEQ-2000’s data was highly consistent with that of NovaSeq6000’s data, with the area under the curve of 1. We also tested the model’s robustness with MGISEQ-2000’s data when reducing the sequencing depth. The results showed that MGISEQ-2000’s data showed matching robustness of the PDACatch classifier with NovaSeq6000’s data.

**Conclusions:**

Taken together, MGISEQ-2000 demonstrated similar data quality, consistency of the methylation levels, comparable analytic sensitivity, and matching clinical performance, supporting its application in future non-invasive early cancer detection investigations by detecting distinct methylation patterns of cfDNAs.

**Supplementary Information:**

The online version contains supplementary material available at 10.1186/s13148-023-01543-4.

## Background

The development of next-generation sequencing (NGS) largely reduced the cost of genome sequencing, taking biological and medical research into a new era [1]. With rapid evolution over the past two decades, the Illumina NGS Sequencers (HiSeq2500, NovaSeq6000, HiSeqX10, etc.), which are based on principles of bridge amplification and sequencing by synthesis (SBS), have become the most widely used platforms and produced the majority of the publicly available sequencing data [2, 3]. Due to its high throughput and analytic accuracy, NGS has been gradually adapted as a cost-effective tool to clinical applications, such as the diagnosis of hereditary disorders and identification of cancer molecular subtypes, by detecting copy number variation, gene fusions, somatic mutations, etc. [4–6].

Recently, MGI Tech has launched a new series of sequencers (BGISEQ-500, MGISEQ-2000, DNBSEQ-T7, etc.) based on DNA NanoBalls (DNBs) amplification and combined primer anchor synthesis (cPAS) technology [7, 8]. It becomes an alternative for high-throughput sequencing by demonstrating comparable output. Moreover, previous studies have demonstrated that the DNB amplification technology has several benefits compared with bridge PCR amplification: DNB amplification is based on linear amplification where each copy is generated from the original DNA fragment; therefore, it avoids clonal error accumulation and molecular switching of sample barcodes, and reduced coverage bias, particularly in GC-rich regions [9, 10]. Recent comparison studies confirmed that MGI platforms show comparable performance on targeted sequencing [11], whole genome sequencing (WGS) [12–15], whole exome sequencing (WES) [16], RNA-Seq [17], scRNA-Seq [18] and metagenomic sequencing [19] with Illumina sequencers. However, MGI platforms’ performances on sequencing low-complexity libraries such as DNA methylation remain to be assessed.

DNA methylation plays a crucial role in modulating various physiological and pathological processes [20]. Many studies have revealed that the abnormal DNA methylation patterns of circulating tumor DNA (ctDNA) are related to cancer pathogenesis and progression, making them promising molecular biomarkers for clinical non-invasive cancer detection [21–23]. However, the ctDNA comprises only a small fraction of total cell-free DNA (cfDNA) during early cancer stage (< 1%), making it difficult to be detected [24]. Bisulfite sequencing is the gold-standard technique that enables quantitative detection of DNA methylation at a single base-pair resolution [25]. Nevertheless, there are some limitations to apply bisulfite sequencing in clinical non-invasive diagnosis. The bisulfite reaction often triggers DNA degradation, which largely diminishes its performance in clinical test [24, 26]. To address this issue, several targeted bisulfite-sequencing technologies have been developed to efficiently capture and amply the signal of targeted regions, for example, MethylTitan [21] and ELSA-Seq [22]. Additionally, the bisulfite-converted libraries typically have low sequence diversity, leading to low data outputs, poor sequencing quality, and high sequencing errors. To improve sequencing quality, a control library is required to balance the base composition [27, 28]. Thus, it is necessary to evaluate a bisulfite-sequencing assay’s sequencing quality and performance before being applied in clinical test. Currently, bisulfite-sequencing assays are developed and validated mainly on Illumina sequencing platforms but have not been thoroughly tested on MGI platforms despite their advantages in the underlying DNB technology.

In this study, we tested the MGI sequencer MGISEQ-2000 on performing targeted bisulfite sequencing using PDACatch, noninvasive assay for pancreatic cancer detection [29]. Synthetic and clinical cfDNA samples were sequenced to evaluate sequencing quality, the consistency of measuring methylation levels, the sensitivity in detecting cancer signals, and the accuracy of PDAC classification, all of which were benchmarked by Illumina’s NovaSeq6000.

## Results

### MGISEQ-2000 showed good sequencing performance on targeted bisulfite sequencing

**Fig. 1:**
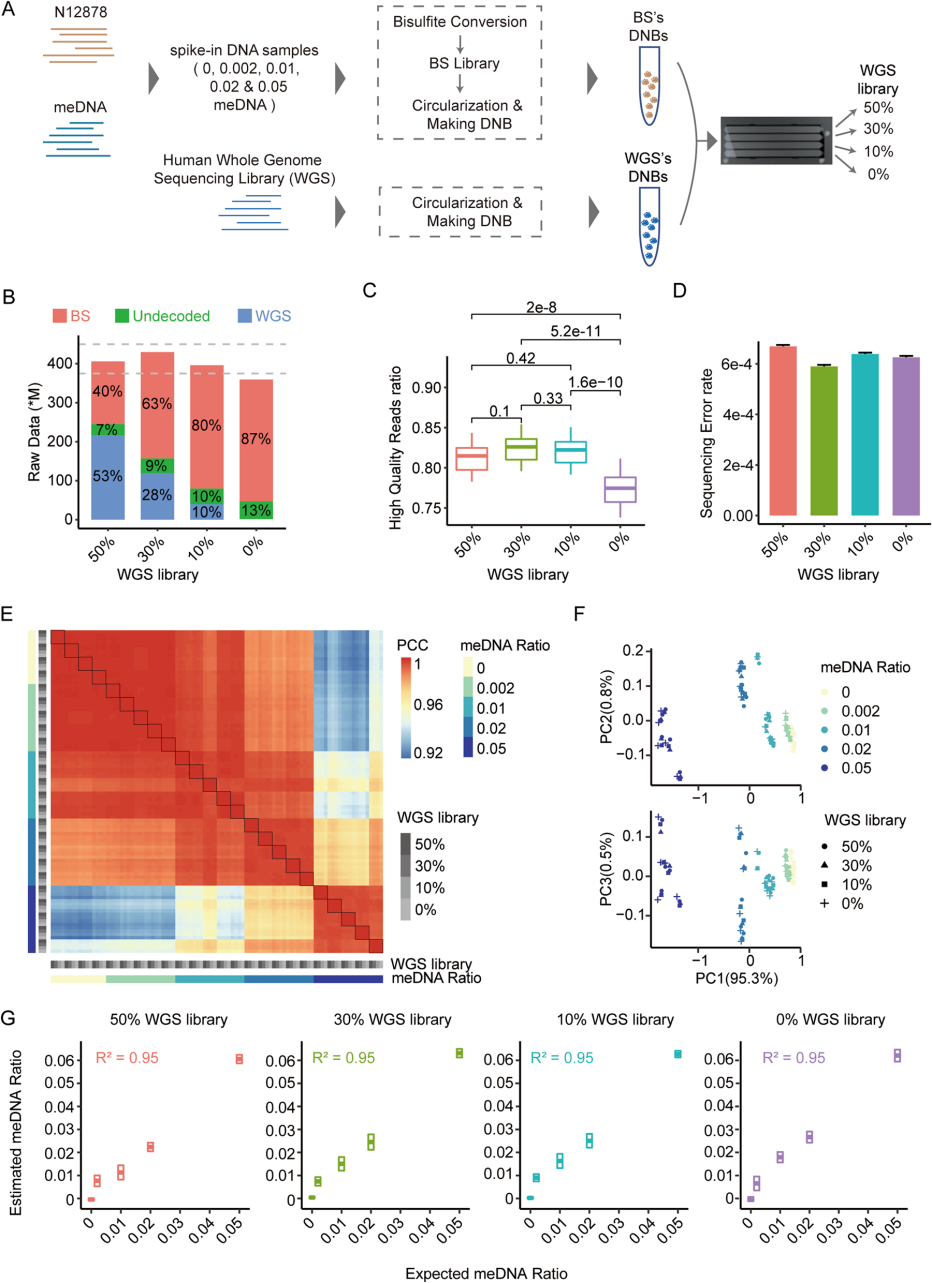
MGISEQ-2000 exhibiting good performance on targeted bisulfite sequencing. **A** The experimental design for testing the sequencing quality of MGISEQ-2000 with different control library contents on targeted bisulfite sequencing. **B** The yielding data output of MGISEQ-2000 with different WGS library contents. Filled colors represented the library types. The dashed lines represented the proposed output interval. **C** The high-quality sequence ratios of methylation library. The sequence of Phred quality score > 30 was defined as the high-quality sequence. The statistical analysis was performed by “Wilcox. test” and adjusted by “holm”. **D** The sequencing error rate of BS data generated with different WGS library contents. The *x*-axis represented the different WGS library contents, the *y*-axis represented the sequencing error rate of BS data, and the error bars depicted the sequencing error ± 95% CI. A base was defined as sequencing error base when the Phred score of the base > 30 and the base in Reads1 was not correctly complementary with Reads2. **E** High correlation of average methylation fractions among replicates. The filled colors represented the Pearson correlation coefficient, and the annotation colors represented the dilution ratios and the percentages of the balance library. The data in a little rectangle represented the same library that was sequenced with different WGS contents, the data in a large rectangle represented the libraries of the same dilution ratio. **F** PCA of average methylation fractions of simulated samples. Colors represented the dilution ratios and shapes represented the percentage of the balance library. **G** The estimated ratios were highly consistent with the expected values. Cross bars depicted the estimated ratio ± 95% CI

To obtain high-quality sequencing data, it was essential to determine the appropriate content of control library for bisulfite sequencing on the MGISEQ-2000 platform, given the impact of unbalanced base compositions on sequencing data outputs and quality [27, 28, 30]. Without a universal control library provided by MGI Tech, we prepared a human whole genome sequencing (WGS) library as the control for following bisulfite sequencing. To generate our targeted bisulfite-sequencing (BS) libraries, we diluted fully methylated genomic DNA (meDNA) into human genomic DNA from a B lymphoblast cell line (NA12878) at the ratios of 0, 0.002, 0.01, 0.02, and 0.05 and prepared the libraries using the MethylTitan protocol [29]. The BS libraries were then sequenced on MGISEQ-2000 (150-bp paired-end) with a decreased percentage of spiked-in WGS libraries (50%, 30%, 10%, and 0%) on four separate lanes of a flow cell (Fig. 1A).

The total number of reads generated by the MGISEQ-2000 was consistent with our expectations of 375 million reads per lane, with a and ‘WGS’ data based on distinctive barcodes, leaving the reads with unknown barcodes as “undecoded”. However, approximately 10% of data could not be exactly decoded and the ratio of undecoded data decreased along with more control library added (Fig. 1B). As for the sequencing quality, we found a significant decrease in the percentage of high-quality reads (with a Phred score ≤ 30) in the data generated with 0% WGS library when compared to others (Fig. 1C, Table 1). The detailed base quality scores also demonstrated a slight decrease in the 0% WGS data (Additional file 1: Fig. 1). Additionally, we defined the sequencing error bases as those whose Phred scores were larger than 30 in both read1 and read2 and were not reverse complemented, and calculated sequencing error rate for each BS library. Results showed that the error rate of bisulfite-sequencing reads was about 6.0 $\times \hspace{0.17em}$10^–4^. Furthermore, the bisulfite-sequencing data generated with a 30% WGS library demonstrated a slightly lower sequencing error rate compared to the data produced with other WGS library contents (Fig. 1D).

**Table 1:** The summary QC of the data of MGISEQ-2000

WGS library ratio (%)	Total data (*M*)	BS Data (*M*)	High-quality reads ratio (%)	Mapping ratio (%)	On-target ratio (%)	Uniformity ratio (%)
50	405.96	160.38	78–84	50–61	72–84	55–59
30	429.89	272.31	80–85	50–62	72–84	55–59
10	396.09	315.93	79–85	50–61	72–84	55–59
0	359.59	312.14	74–81	50–61	72–84	55–58

“WGS library ratio” represented the percentage of spike-in WGS library. “Total Data” and “BS Data” represented total data yield and bisulfite-sequencing data yield with the different contents of WGS library, respectively. “High-quality reads ratio” demonstrated the ratio of high-quality reads (phred > 30) in BS data. “Mapping Ratio” represented the ratio of BS reads which could be aligned to human genome. ‘On-target Ratio’ represented the ratio of mapping reads which were amplified by panel primers and located in targeted genome regions. ‘Uniformity Ratio’ demonstrated the uniformity of panel targeted priming which was calculated using the ratio of CpGs whose coverages were larger than 25% median coverage. The values before “–” represented the minimum values, while those after “–” represented the maximum values

Furthermore, we evaluated the consistency of the average methylation fractions (AMFs, see “Methods” section for definition) of targeted regions across all sequenced BS libraries. Our findings demonstrated a high pairwise correlation coefficient of 0.999 between BS libraries of different spiked-in WGS control contents, indicating a very high concordance among them (Fig. 1E). We also performed principal component analysis (PCA) on the AMFs and found that the projections of PC1, which accounted for 95.3% of libraries variances, were along with meDNA fractions, while the variances of PC2 (0.8% variance) and PC3 (0.5% variance) primarily reflect library preparation deviations (Fig. 1F). This result suggested that the variance of sequencing was minor compared to that of library preparation. Furthermore, we evaluated the quantitative accuracy of bisulfite sequencing on MGISEQ-2000 with different control library contents. The results showed that the estimated meDNA ratios were well correlated with the expected ratios (*R*^2^ = 0.95) in the four datasets generated with different control library contents (Fig. 1G). We also found that the estimated meDNA ratios were slightly higher than the expected one, suggesting that the MGISEQ-2000 might detect higher methylation levels than anticipated (Fig. 1G). Overall, these results suggested that the balanced control library content primarily affected the data outputs, sequencing quality, and sequencing error rate, but had a negligible impact on the consistency and quantitative precision of DNA methylation levels.

### MGISEQ-2000 showed similar data quality with NovaSeq6000

**Fig. 2:**
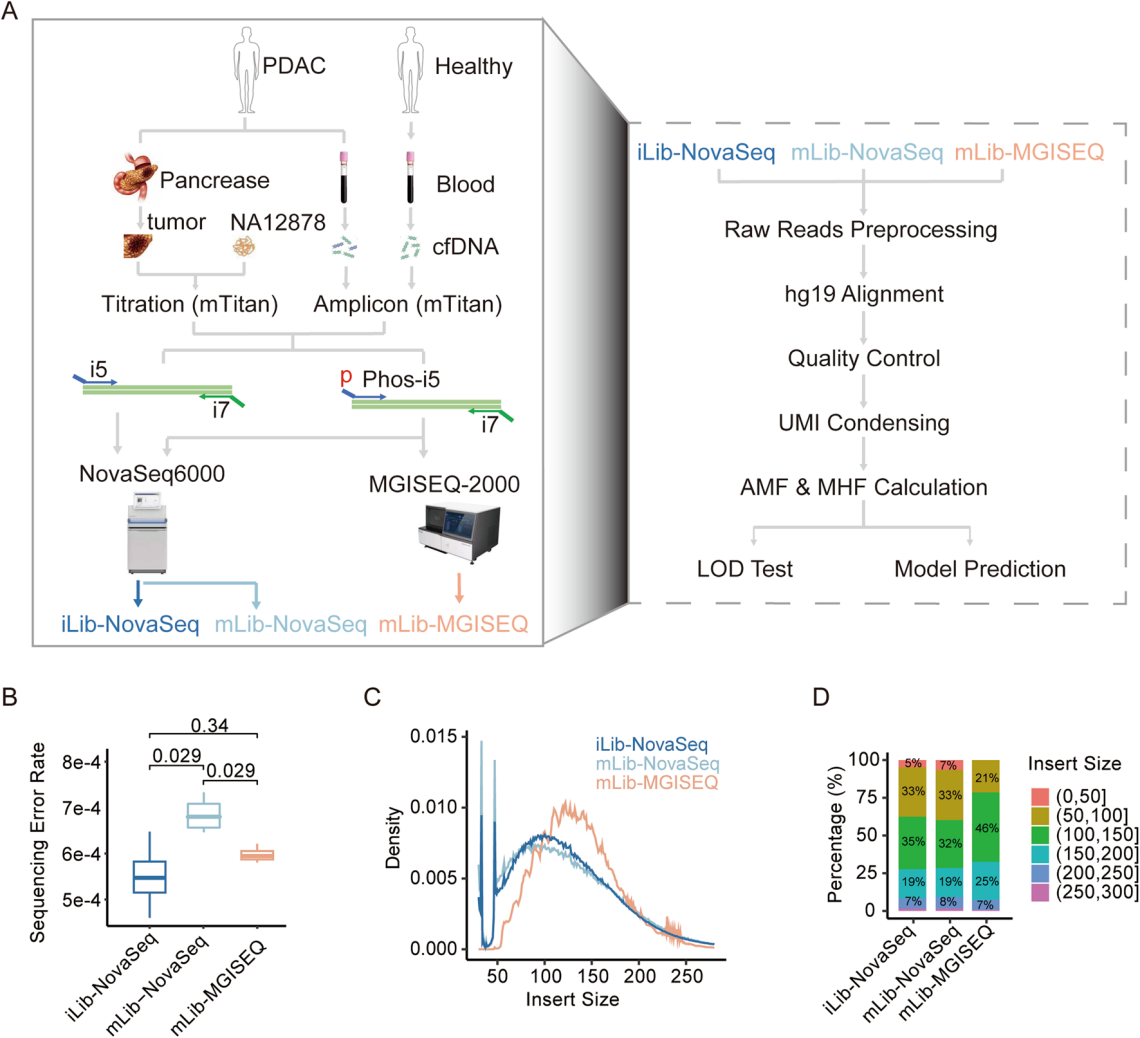
MGISEQ-2000 showed similar data quality with NovaSeq6000. **A** Design of cross-platform comparison. In brief, we compared the targeted methylation sequencing of NovaSeq6000 and MGISEQ-2000 with the synthetic cfDNA samples and clinical cfDNA samples. The synthetic cfDNA samples were made by spiking pancreatic ductal adenocarcinoma (PDAC) gDNA into NA12878 at tumor fractions of 0, 0.1%, 0.5%, 1%, 5%, and 10% (four replicates for each tumor fraction). We also used 24 cfDNA samples, which were from 12 PDAC patients and 12 healthy donors. Two libraries were prepared with Illumina official experimental kits and MGI official experimental kits, and renamed as “iLib” and “mLib”. The iLib were sequenced by NovaSeq6000 and mLib were sequenced by NovaSeq6000 and MGISEQ-2000, which were finally allocated to three data ‘iLib-NovaSeq’, ‘mLib-NovaSeq’ and ‘mLib-MGISEQ’. The analysis pipeline was shown on the right panel. **B** Boxplot plot showing the sequencing error rate of iLib-NovaSeq, mLib-NovaSeq, and mLib-MGISEQ data. The statistical analysis was performed by “Wilcox. test” and adjusted by "holm". **C** The distribution of insert size of sequencing data of the synthetic cfDNA samples. The *x*-axis represented the insert size of alignments, the *y*-axis represented the density of insert size distribution; colors represented data types. **D** The percentage of alignments in different insert size intervals. We made 50 bp-bin intervals and summarize the percentage of alignments in the intervals. Filled colors represented the intervals of insert size

To determine whether MGISEQ-2000 has comparable performances as mainstream sequencing platforms in DNA methylation sequencing, we conducted a head-to-head cross-platform comparison between the MGISEQ-2000 and the Illumina NovaSeq6000 sequencer (Fig. 2A). We prepared a series of synthetic cfDNA samples as test sample to reduce experimental variations between the sequencers. The synthetic cfDNA samples were generated by diluting the pancreatic ductal adenocarcinoma (PDAC) genomic DNA (gDNA) into NA12878 at tumor fractions of 0%, 0.1%, 0.5%, 1%, 5%, and 10%. To minimize influence of reagents, we prepared two sets of libraries, “iLib” and “mLib”, using official Illumina and MGI library preparation kits, respectively. Note that the “mLib” libraries can be sequenced on both MGISEQ-2000 and NovaSeq6000. The quality and length distribution of the sequencing libraries were checked through LabChip GX, and results demonstrated that the mLib and iLib had nearly identical curves, the overall libraries length was around 200–500 bp, which showed those sequencing libraries had the same size distribution (Additional file 2: Fig. 2A). Then, the “iLib” libraries were sequenced on NovaSeq6000, while the “mLib” were sequenced on both NovaSeq6000 and MGISEQ-2000, which resulted in three data sets: iLib-NovaSeq, mLib-NovaSeq, and mLib-MGISEQ.

Our analyses showed that the data quality was similar among the three data sets, as demonstrated by key quality control parameters (Table 2). The sequencing error rates of mLib-MGISEQ were comparable to those of iLib-NovaSeq but were significantly lower than mLib-NovaSeq, suggesting that it was better to prepare and sequence libraries using library preparation kits and sequencers produced by the same manufacturer (Fig. 2B). Moreover, we observed a great difference in the inserts’ size distribution between mLib-MGISEQ and iLib-NovaSeq as previously reported [11] (Fig. 2C). The mLib-MGISEQ dataset showed a significant loss of 50–100 fragments, which comprised of 21% of total mLib-MGISEQ data, compared to 33% in iLib-NovaSeq and mLib-NovaSeq (Fig. 2D). Because the mLib shared the same library size with the iLib, we speculate that the loss of short fragments by MGISEQ-2000 may be due to the library circularization step during making DNBs, which may limit the application of MGISEQ-2000.

**Table 2:** The summary QC of the data of MGISEQ-2000 and NovaSeq6000

Data type	Library type	Sequencer	High-quality reads ratio (%)	Mapping ratio (%)	On-target ratio (%)	Uniformity ratio (%)
mLib-MGISEQ	mLib	MGISEQ-2000	86–88	46–49	75–79	56–60
iLib-NovaSeq	iLib	NovaSeq6000	86–89	49–52	70–75	59–64
mLib-NovaSeq	mLib	NovaSeq6000	86–88	48–52	72–75	59–64

“Library Type” represented the kit used to prepare libraries. “Sequencer” represented the sequencer to generate data. High-quality reads ratio” demonstrated the ratio of high-quality reads (phred > 30). “Mapping Ratio” represented the ratio of reads that aligned to human genome. ‘On-target Ratio’ represented the ratio of mapping reads which were amplified by panel primers and located in targeted genome regions. ‘Uniformity Ratio’ demonstrated the uniformity of panel targeted priming, which was calculated using the ratio of CpGs whose coverages were larger than 25% median coverage. The values before “–” represented the minimum values, while those after “–” represented the maximum values

### MGISEQ-2000 showed highly consistent methylation levels with NovaSeq6000

**Fig. 3:**
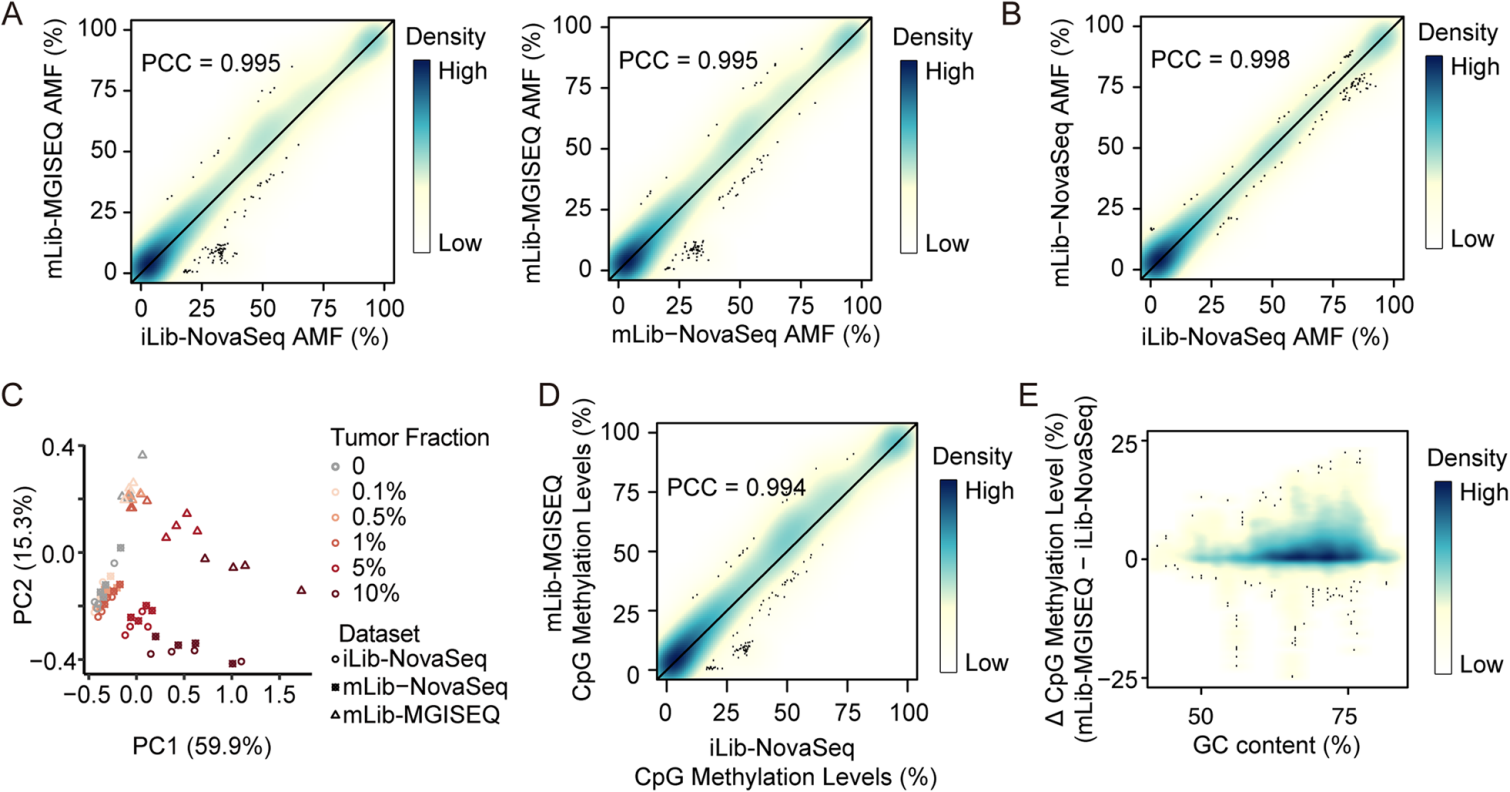
MGISEQ-2000 showed consistent methylation levels with NovaSeq6000. **A**, **B** High correlation of AMFs between iLib-NovaSeq and mLib-MGISEQ (left of A), mLib-NovaSeq and mLib-MGISEQ (right of A), and iLib-NovaSeq and mLib-NovaSeq (B). Color represented the density of points. Points represented the outliers. The black line represented *y* = *x*. **C** PCA of AMFs of the synthetic cfDNA samples. Colors represented the dilution ratios, and shapes represented the data types. **D** High correlation of CpG methylation levels between iLib-NovaSeq and mLib-MGISEQ. **E** The variation of CpG methylation levels between mLib-MGISEQ and iLib-NovaSeq datasets around GC contents. The GC content of a CpG site was calculated in a 200-bp window which were extended upstream 100 bp and downstream 100 bp of the CpG

Next, we compared methylation levels measured by the mLib-MGISEQ and iLib-NovaSeq datasets. Our analysis indicated that the mLib-MGISEQ and iLib-NovaSeq datasets demonstrated remarkably high level of consistency in the AMFs with a Pearson's correlation coefficient (PCC) of 0.995, which was only slightly lower than that between the data of iLib-NovaSeq and mLib-NovaSeq (PCC = 0.998, Fig. 3A, B). However, we also detected systematic discrepancies between the two sequencing platforms. The PCA on the AMFs revealed that the variance of PC1 (59.9%) was mainly associated with the tumor fractions, while that of PC2 (15.3%) corresponded to the systematic discrepancy between NovaSeq6000 and MGISEQ-2000 (Fig. 3C). Then, we selected the top 5% variable regions related to systematic discrepancy and defined them as highly variable regions (Additional file 1: Fig. 3A). The GC contents of these regions were significantly higher than those of random selected regions, indicating that the inter-sequencer variation was related to GC contents of the local regions (Additional file 1: Fig. 3B).

We further analyzed the systematic discrepancies between MGISEQ-2000 and NovaSeq6000 on CpG sites. The result showed that the CpG methylation levels of MGISEQ-2000 were good accordance with those of NovaSeq6000 with a PCC of 0.994 (Fig. 3D). However, MGISEQ-2000 measured higher methylation levels than NovaSeq6000 in the CpG sites with high GC contents (Fig. 3E). Besides, 26.3% of detected CpG sites, which were discordantly methylated (methylation ratios were between 0.2 and 0.8), showed higher methylation levels by MGISEQ-2000 than NovaSeq6000 (Additional file 1: Fig. 3C). MGISEQ-2000 showed more consistency with NovaSeq6000 in un- to lowly methylated CpGs (0–20%) or highly methylated CpG (80–100%). When compared the methylation levels of CHN site, we also found MGISEQ-2000 measured higher methylation levels (Additional file 1: Fig. 3D). Since iLib and mLib came from the same bisulfite conversion libraries, we hypothesized that MGISEQ-2000 might detect higher false-positive methylation levels than NovaSeq6000.

### MGISEQ-2000 showed comparable cancer signal detecting ability compared with NovaSeq6000 at the tumor fraction of 0.1%

**Fig. 4:**
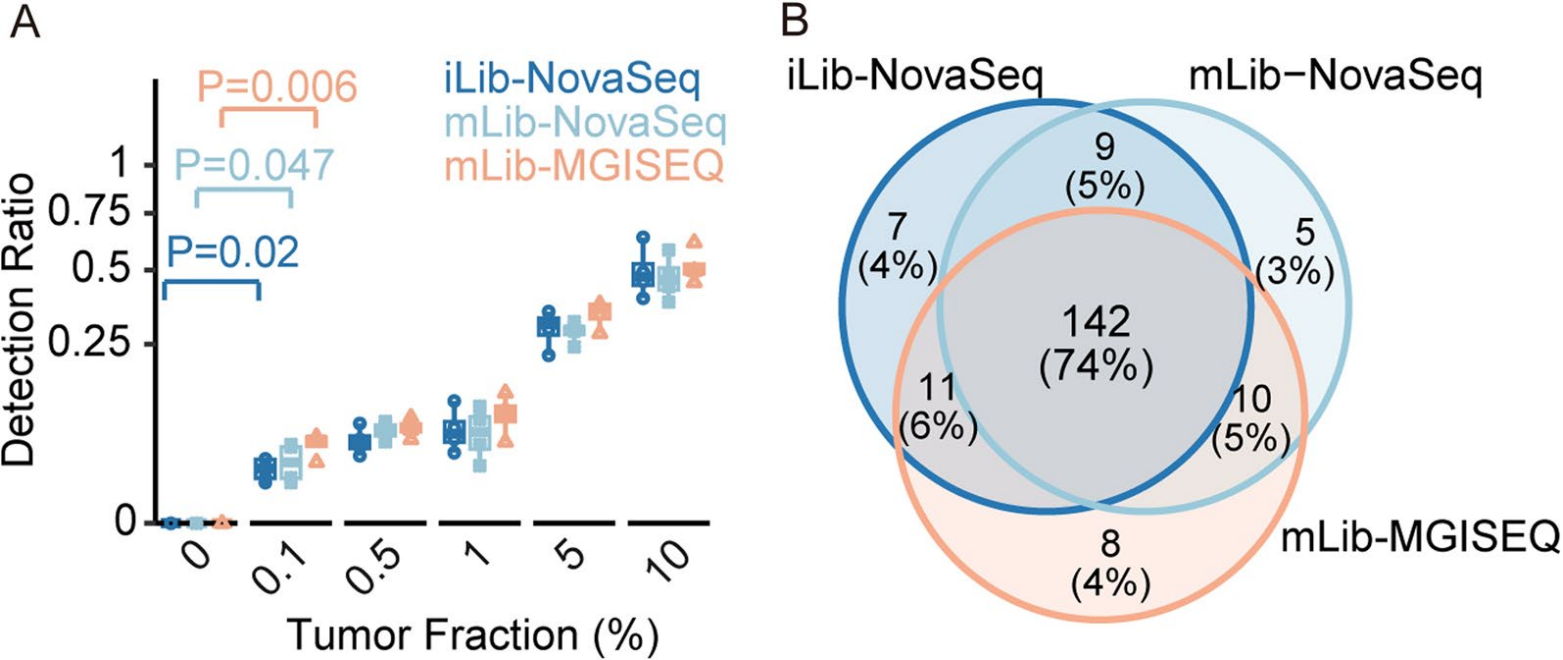
MGISEQ-2000 showed comparable cancer signal detecting ability with NovaSeq6000 at the tumor fraction of 0.1%. **A** The limit of detection of iLib-NovaSeq, mLib-NovaSeq, and mLib-MGISEQ. The *y*-axis represented the percentage of observed positive markers. Colors represented data types. There were four replicates at each tumor fraction. Statistical analysis was performed by a two-sided *t*-test. **B** The Venn plot showing the most of detected markers was detected in the three types of data. The markers, which were detected in two replicates at the tumor fraction of 0.1, were defined as detected markers

The sensitivity of an assay is important for its clinical applications. To determine the assay’s sensitivity on different sequencers, we performed an LOD analysis. We calculated detection ratios (the ratios of detected markers) of iLib-NovaSeq, mLib-NovaSeq, and mLib-NovaSeq data at different tumor fractions to represent the detected cancer signals (seeing methods for detail). Notably, our results revealed that MGISEQ-2000 could also significantly detect cancer signals at the tumor fraction of 0.1% as NovaSeq6000 (Fig. 4A). The results suggested that the systematic discrepancy did not impair the sequencer’s detection ability.

Moreover, we defined the markers which were detected in two replicates of synthetic cfDNA samples at the tumor fraction of 10% (four replicates for each dataset) as detected markers. The results highlighted that 74% of the detected markers were shared in the datasets of iLib-NovaSeq, mLib-NovaSeq, and mLib-MGISEQ, while only 5% of the detected markers were batch-effect (Fig. 4B). These findings suggest that MGISEQ-2000 has a comparable sensitivity in detecting cancer signals as NovaSeq6000, demonstrating its potential for clinical application.

### MGISEQ-2000 showed matching clinical performance compared with NovaSeq6000

**Fig. 5:**
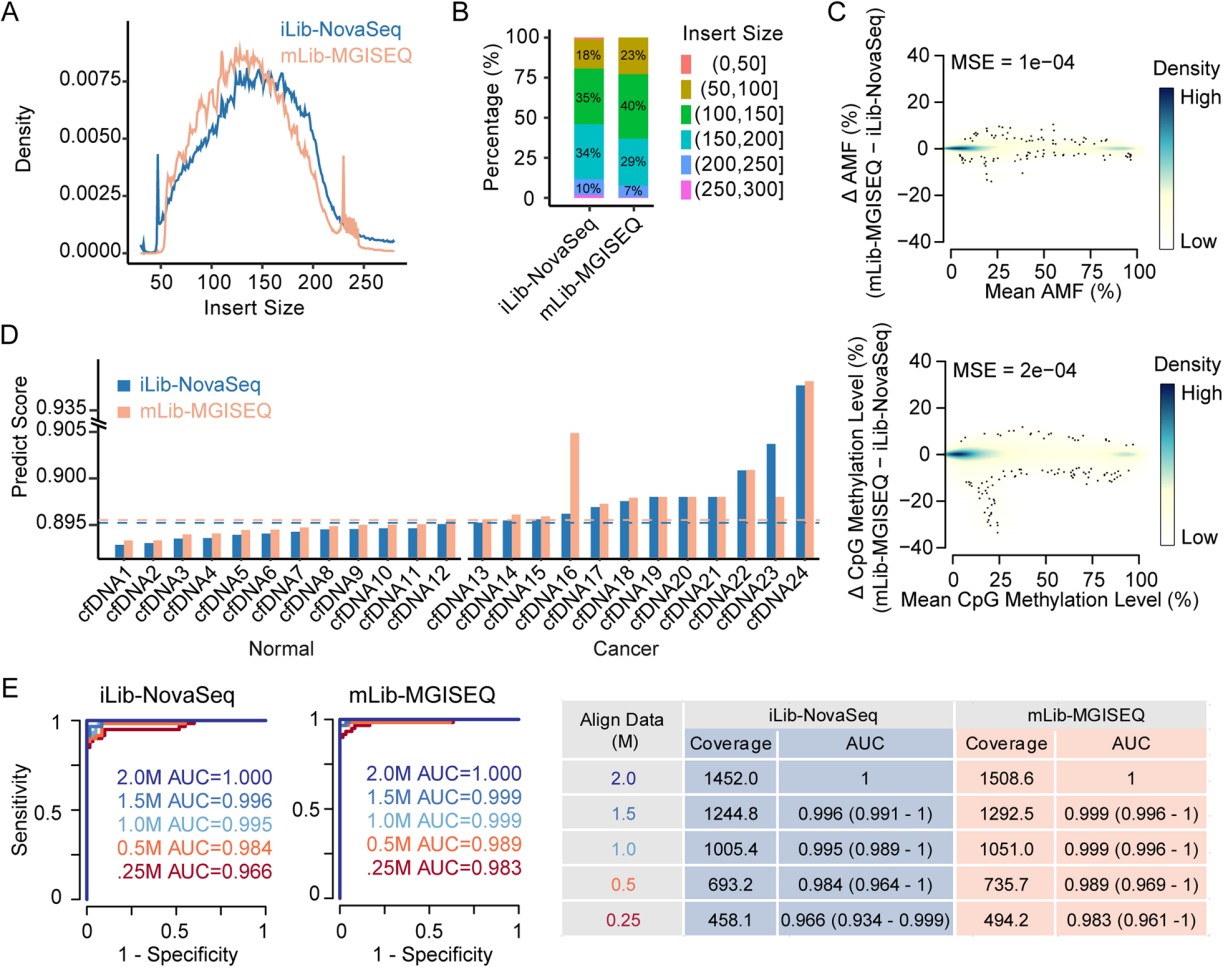
MGISEQ-2000 exhibited matching clinical performance with NovaSeq6000. **A** The distribution of insert size of sequencing data of clinical cfDNA samples. The *x*-axis represented the insert size of sequencing data; the *y*-axis represented the distribution of insert size; colors represented different sequencers. **B** The percentage of alignments in different insert size intervals. Filled colors represented the intervals of insert size. **C** Scatter plot showing minor variances of AMFs (upper) and CpG methylation levels (below) of mLib-MGISEQ and iLib-NovaSeq. The *y*-axis represented the deviation values of mLib-MGISEQ and iLib-NovaSeq, and the *x*-axis represented the mean values. Colors represented the density of points. Black points represented the outliers. MSE represented the mean squared error and bias represented the mean error. **D** The prediction scores of 24 cfDNAs. The *x*-axis represented the 24 cfDNAs; the *y*-axis represented the prediction score with the in-house model; the filled colors represented sequencers. The prediction scores of mLib-MGISEQ were positively correlated with those of iLib-NovaSeq. The blue dashed line represented the threshold of 0.8952 for NovaSeq6000 data and the orange dashed line represented the threshold of 0.8956 for MGISEQ-2000 data. **E** The receiver operating characteristic curves (ROCs) of down-sampling iLib-NovaSeq6000 data (left), and mLib-MGISEQ-2000 data (middle). The colors on the left and middle panels represented the size of the down-sampling data. The table on the right represented the down-sampling data sizes and their corresponding coverages and area under curves (AUCs) and the numbers in brackets represented AUCs ± 95% CI

To assess the performance of MGISEQ-2000 on clinical samples, we sequenced 24 cfDNA samples (12 samples from healthy donors and 12 samples from PDAC patients) purchased from ProteoGenex, a commercial biobank. We generated “iLib” and “mLib” for each cfDNA sample and sequenced them on NovaSeq6000 and MGISEQ-2000, respectively (Fig. 2A). The size distribution of iLib and mLib libraries was similar on Labchip GX (Additional file 1: Fig. 2B), the overall fragment length for clinical cfDNA libraries was around 200–600 bp and the main peak located nearly 320 bp. Interestingly, the insert size of the mLibs was comparable to that of iLibs, which was differed from the results when synthetic cfDNA samples were sequenced (Fig. 5A, B).

We also compared the systematic bias on the overall AMF values of targeted regions, and methylation levels of individual CpG site in the targeted regions between the data of mLib-MGISEQ and iLib-NovaSeq. We found that the Pearson’s correlation coefficients of AMF and CpG sites’ methylation levels were 0.999 and 0.998, respectively. We also calculated the deviation of AMFs and CpG sites’ methylation levels between the corresponding replicates, which were 1e-4 and 2.3e-4 Mean Squared Error (MSE) for AMFs and CpG methylation levels, respectively. This indicated a minimal systematic discrepancy between NovaSeq6000 and MGISEQ-2000 in targeted bisulfite sequencing of cfDNA (Fig. 5C). However, as for the CpG sites with methylation ratios between 0.2 and 0.6, the data generated by MGISEQ-2000 detected slightly higher methylation levels than those generated by NovaSeq6000 (Additional file 1: Fig. 3E). MGISEQ-2000 also detected higher methylation levels on CHN sites, suggesting that the methylation levels of mLib-MGISEQ might exhibit a higher false-positive rate (Additional file 1: Fig. 3F).

We next compared the performances of a pre-built PDACatch classifier on NovaSeq6000 and MGISEQ-2000 data. The PDACatch classifier was used to predict and distinguish ctDNA samples of PDAC patients from healthy individuals based on the prediction scores and has been validated in real-world clinical samples [29]. Here, the PDACatch prediction scores for each test sample were calculated by the same formula using methylation levels of the PDACatch’s targets measured by either mLib-MGISEQ or iLib-NovaSeq as variables. Of the 24 cfDNA samples tested, only two, cfDNA16 and cfDNA23, had drastically different prediction scores between mLib-MGISEQ and iLib-NovaSeq (Fig. 5D), while the rest were very similar. Indeed, Pearson correlation analysis on these scores showed a high correlation coefficient of 0.971 (Fig. 5D). Moreover, we were able to classify test PDAC and healthy cfDNA samples at 100% accuracy using two highly similar thresholds, 0.8952 for iLib-NovaSeq data and 0.8956 for mLib-MGISEQ. These results demonstrated a high consistency of PDAC classification between mLib-MGISEQ and iLib-NovaSeq (Fig. 5D) despite a small degree of discrepancy that need to be adjusted cross-platform-wise. Lastly, we down-sampled the aligned data from either platform to 2.0 M, 1.5 M, 1 M, 0.5 M, and 0.25 M, respectively, and used the down-sampled data to classify test samples to compare the robustness of the PDCA classifier. Interestingly, we found the PDACatch classifier performed more robustly using mLib-MGISEQ dataset than iLib-NovaSeq (Fig. 5E), as its AUC scored decreased at a slower pace than NovaSeq600. Taken together, the comparison on the performances of PDACatch classifier on MGISEQ-2000 and NovaSeq6000 platforms suggested that while it was developed by NovaSeq6000 platform, PDACatch performed accurately and robustly using MGISEQ-2000 data with minor adjustment on the classification threshold. This suggests that it may be possible to maintain a classifier’s performances that was initially developed on an Illumina platform after switching to a MGISEQ-2000 sequencer, where only minimal classifier re-training is needed.

## Discussion

In this study, we tested MGISEQ-2000 on targeted bisulfite sequencing to assess its potential application. We benchmarked MGISEQ-2000’s performances by conducting a head-to-head comparison NovaSeq6000, the widely used sequencer for methylation sequencing; the results showed that MGISEQ-2000 demonstrated similarly high quality of raw data, consistent methylation levels, comparable detection sensitivity and similar accuracy in classifying clinical samples with NovaSeq6000. Together, these results strongly suggest MGISEQ-2000 has the potential to be applied to detect and measure DNA methylation changes clinical tests, especially cfDNA methylation markers.

Previous studies have reported that MGI platforms showed consistent performances with Illumina platforms on transcriptomes, WGS, metagenomics, among others [13–19]. However, so far, no study has been reported about testing MGI sequencers on DNA methylation. In this study, we first tested the MGISEQ-2000 on DNA methylation sequencing with different levels of balancing library, whose results showed that the inadequate level of balancing library mainly reduces high-quality data and sequencing accuracy, while having little impact on calling methylation levels. Considering the cost and data quality, we propose to use 10–30% of balancing library for bisulfite sequencing on MGISEQ-2000, while the exact percentage may need more tests. We also found that MGISEQ-2000 might measure higher methylation levels than NovaSeq6000 at CHN sites and at CpG sites with methylation ratios between 0.2 and 0.8. Given that the sequencing libraries were made from aliquots of the same bisulfite-converted DNA samples, we hypothesized that MGISEQ-2000 may produce slightly artificially elevated methylation levels compared to NovaSeq6000. However, as the absolute methylation levels in the DNA standards were unknown, this hypothesis requires further investigation.

To evaluate the MGISEQ-2000’s performance in sequencing clinical samples, we not only tested its technical sensitivity with synthetic cfDNA samples of different levels of spiked-in cancer DNA, but also used it to classify 24 clinical samples (12 healthy and 12 PDAC plasmas) using the PDACatch classifier in a cross-platform comparison. Though PDACatch was initially developed and validated on Illumina platforms, these samples had very similar PDACatch scores based on methylation data from either MGISEQ-2000 or NovaSeq6000. On the other hand, our findings revealed that the cutoff of the PDACatch classifier was indeed slightly different between the two platforms. However, given the small sample size (12 normal and 12 patients), the training models are prone to be overfitted in the tenfold cross-validation, making large random variance and sampling bias. Taken together, we concluded that, based on our preliminary results, the cutoff of a classifier may need minor adjustment to ensure consistent classification outcomes between MGISEQ-2000 and Illumina platforms such as NovaSeq6000. This is essential for MGISEQ-2000 and its sister sequencers to be applied clinically, and more clinical samples need be tested on MGI sequencers to validate this conclusion.

Recently, several studies have revealed the fragmentomics and ultra-short fragments of cfDNA are also important biomarkers in clinical cancer detection [31–36]. Thus, it should be noted that our preliminary results showed that the data of MGISEQ-2000 had a loss of the ultra-short fragments, in which the fragment size was around 50 bp, compared to NovaSeq6000. Circularizing oligonucleotides becomes more challenging for ultra-short fragments because of increased bending rigidity caused by shortened length. As a result, researchers may need to use specialized techniques such as using longer adapters to increase circularization efficiency to retain these ultra-short fragments [37]. Moreover, single- and double-stranded DNA species of different sizes, such as a ladder, may be spiked into libraries prior to sequencing to measure the variances in size retention between the two sequencers. This will allow the comparison of the accuracy and reliability of the two sequencers in determining the exact sizes of sequenced DNA fragments, which is essential to identify the suitable sequencer to study and translate fragmentomics features of cfDNA into clinical applications. Due to the relatively small number of samples tested in our study, we cannot rule out that random variations in library preparation and sequencing caused this discrepancy in size between MGISEQ-2000 and NovaSeq6000. Therefore, additional investigation is needed to carefully interrogate whether MGISEQ-2000-based library preparation and sequencing procedures indeed favor larger fragments [11]. Results from this investigation will be important to determine the suitable sequencer to study and translate fragmentomics features of cfDNA into clinical applications.

## Conclusions

In summary, we conducted targeted bisulfite sequencing on MGISEQ-2000 and found that it demonstrates similar sequencing quality, consistent methylation levels, comparable technical sensitivity, and matching clinical model performance with NovaSeq6000, supporting its application in future noninvasive early cancer detection investigations by motoring DNA methylation changes. Our findings may also apply to other clinical assays based on DNA methylation.

## Methods

### The design of cross-platform comparison

To assess the clinical application of MGISEQ-2000 sequencer on bisulfite sequencing, we evaluated the data quality, methylation calling consistency, the sensitivity in detecting cancer signal and clinical accuracy, with NovaSeq6000 as the benchmark. The comparison was performed with synthetic cfDNA samples and clinical samples (Fig. 2A).

For synthetic cfDNA samples, we diluted the pancreatic ductal adenocarcinoma (PDAC) genomic DNA (gDNA) into NA12878 at tumor fractions of 0%, 0.1%, 0.5%, 1%, 5%, and 10% and generated libraries with the Singlera MethylTitan protocol. For each sample, we prepared two libraries using Illumina official experimental kits (canonical i5 sequencing primers) and MGI official experimental kits (phosphorylated i5 sequencing primers), respectively, and renamed them as “iLib” and “mLib”. Specifically, the “mLib” were sequenced both on NovaSeq6000 and MGISEQ-2000, and the “iLib” were only sequenced on NovaSeq6000.

As for the clinical samples, we also generated “iLib” and “mLib” for each sample. The “iLib” was sequenced on NovaSeq6000, while the “mLib” was sequenced on MGISEQ-2000, respectively.

### Samples preparation

FFPE PDAC tissue and clinical plasma samples (12 preoperative PDAC plasma samples and 12 healthy controls) were purchased from ProteoGenex (Inglewood, CA, USA), seeing detailed in our previous study [29]. cfDNA was extracted using the QIAamp Circulating Nucleic Acid Kit (QIAGEN, 55114), following the manufacture’s recommendations. FFPE tissue gDNA was extracted using Promega Reliaprep FFPE gDNA Miniprep System (Promega, A2352), following the manufacturers’ guidelines. The universal methylated DNA standards and the gold-standard reference samples NA12878 were purchased from Zymo and Coriell, respectively.

Aliquots of 1000 ng of tissue gDNA, methylated DNA standards and reference NA12878 were subjected to fragmentation procedures using the Bioruptor NGS (Diagenode, USA). Briefly, Bioruptor fragmentation was performed with DNA extracts diluted in TE buffer to a final volume of 100 µl and using 20 cycles of 30’’/30’’ (ON/OFF cycles). The products were purified with 1.6 × AMpure XP Beads (Beckman Coulter, A63881).

DNA quality for each extracted sample was measured by evaluating quantity, purity, and fragment length. Samples were quantified using Qubit dsDNA BR Assay (ThermoFisher Scientific, Waltham, MA, USA). The fragment sizes were analyzed with LabChip GX Touch Nucleic Acid Analyzer (PerkinElmer, Hopkinton, Massachusetts, USA).

### Library preparation

For NovaSeq6000 platform-specific library preparation (named as iLib), plasma samples were processed with a standard “mTitan” pipeline [29]. Briefly, the cfDNA was bisulfite-converted using the Methylcode Bisulfite Conversion Kit (ThermoFisher, MECOV50) according to the manufacturer’s protocol. The bisulfite-converted DNA was dephosphorylated and ligated to a universal adapter with a unique molecular identifier (UMI). Following a second-strand synthesis and purification, the DNA underwent a semi-targeted amplification. Following purification, a second PCR-added sample-specific barcodes and full-length sequencing adapters. The libraries were then quantified using the KAPA Library Quantification Kit for Illumina (KK4844). For MGISEQ2000 platform, the purified semi-targeted amplification products were amplified with phosphorylated and unmodified i5 sample-specific barcode primers (named as mLib), the products were further circularized using MGIEasy App-A Kit (MGI, 1000004155) following the manufacture’s recommendations. For NovaSeq6000 platform, the calibration control PhiX library was used to calculate phasing and pre-phasing. For MGISEQ2000 platform, the circularized WGS libraries were used as calibration control.

### Data processing

The base-calling of MGISEQ-2000 data was pre-processed with Zebra call (base calling software developed for MGI sequencers), and the header of the data were re-formatted as Illumina with FastQC (v0.11.7). We assembled the 150 paired-end reads to single-end data using pear (v0.9.6) [38] with the parameters “-j 4 -v 20 -t 30 -n 30”. The adapters and low-quality bases were trimmed by trim_galore (v0.4.0) (https://www.bioinformatics.babraham.ac.uk/projects/trim_galore/) with default parameters. Then, we extracted the UMIs from reads and aligned the reads to hg19 using bismark (v0.17.0) (https://www.bioinformatics.babraham.ac.uk/projects/bismark/) with the parameters “-bowtie2 -l 32 -n 1 -non_directional”. For following analysis, we only kept the on-targeted reads which were with our designed adapters and expected genomic locations and filtered the PCR duplicates according to UMIs using umi_tools (version 1.1.2). Finally, the on-targeted and PCR duplicates removed data were used to generate the following quantitative metrics: the average methylation fractions (AMFs) of targeted regions, the methylation haplotype fractions (MHFs) per candidate haplotype of targeted regions, and the methylation haplotype loads (MHLs) of targeted regions [21, 29]. The formulas are as follows:

$${\text{AMF}} = \frac{{\mathop \sum \nolimits_{i}^{M} N_{C,i} }}{{\mathop \sum \nolimits_{i}^{M} \left( {N_{C,i} + N_{T,i} } \right)}}$$

where *i* represented the index of CpG sites in this target region, *M* was the total number of CpG sites in this target region, $N_{T,i}$ was the number of *T* counted at the *i*th CpG site in this target region, $N_{C,i}$ was the number of *T* counted at the *i*th CpG site in this target region.

$${\text{MHF}}_{i,h} = \frac{{N_{i,h} }}{{N_{i} }}$$

where *i* represented the current locus, *h* represented the current haplotype, $N_{i,h}$ represented the number of reads at the current locus containing the current haplotype, and $N_{i}$ represented the total number of reads covering the current locus.

$${\text{MHL}} = \frac{{\mathop \sum \nolimits_{i = 1}^{l} w_{i} \times P\left( {{\text{MH}}_{i} } \right)}}{{\mathop \sum \nolimits_{i = 1}^{l} w_{i} }}$$

where *l* represented the length of haplotypes and $P\left( {{\text{MH}}_{i} } \right)$ represented the fraction of fully successive methylated CpGs within *i* loci. $w_{i}$ represented the weight for the *i*-locus haplotype. The options for weights were $w_{i}$ = *i* for MHL and $w_{i}$ = *i*^3^ for MHL3.

### LOD analysis

We calculated analytical limit of detection (LOD) as previously reported [22, 39]. We made dilution samples by spike the gDNA of FFPE PDAC tissues into NA12878 at the tumor fractions of 0.1%, 0.5%, 1%, 5% and 10%, and the mock spike-in samples were set as 0. The experiments were repeated four times. We defined the mock dilution samples (tumor fraction of 0%) as baseline samples and trained the baselines of AMFs for each target region. If the AMF of a target region of a sample was out of the baselines, the target region was determined as a detected marker in the sample. Then, we calculated the detection ratio using the count of detected markers divided the count of total markers. The formula is as follows:

$${\text{Baseline}}\;L_{i} = \mu_{i} - 3*{\text{sd}}_{i}$$

$${\text{Baseline}}\;H_{i} = \mu_{i} - 3*{\text{sd}}_{i}$$

$$\mu_{i} = \sum\nolimits_{k = 1}^{N} {{\text{AMF}}_{i,k} /N}$$

$${\text{sd}}_{i}^{2} = \frac{1}{N - 1} \times \mathop \sum \limits_{k = 1}^{N} \left( {{\text{AMF}}_{i,k} - \mu_{i} } \right)$$

$${\text{Detect}}_{{i,j}} = \left\{ {\begin{array}{*{20}l} {1,~\;{\text{AMF}}_{{i,j}} \;\left\langle {{\text{Baseline}}\;L_{i} \;{\text{or}}\;{\text{AMF}}_{{i,j}} } \right\rangle \;{\text{Baseline}}\;H_{i} } \hfill \\ {0,\;{\text{Baseline}}\;L_{i} \le {\text{AMF}}_{{i,j}} \le {\text{Baseline}}\;H_{i} } \hfill \\ \end{array} } \right.$$

$${\text{DetectRatio}}_{j} = \mathop \sum \nolimits_{i = 1}^{M} {\text{Detect}}_{i,j} /M$$

where *i* represented the index of the targeted regions, *k* represented the index of baseline sample, *j* represented the index of dilution samples, *N* represented the count of the baseline samples, *M* represented the count of the total markers.

### The quantitative precision

We estimated the quantitative precision of the MGISEQ-2000 sequencer. The sequencing data were generated by the dilution samples, which were produced by mixing the universal methylated DNA standards (Zymo, D5014-2) and the gold standard reference samples NA12878 (Coriell) at the predefined ratios of 0.002, 0.01, 0.02 and 0.05 (five replicates per ratio). The mock dilution samples (water) were defined as 0 (four replicates). Then, we evaluated the quantitative precision using the expected spike-in ratios compared to the expected dilution ratios. The estimated spike-in ratio of the dilution sample was calculated as the mode of estimated fractions of all target regions in the dilution sample.

$$F_{i,j} = \left( {{\text{AMF}}_{i,j} - \mu_{i} } \right)/\left( {1 - \mu_{i} } \right)$$

$$\mu_{i} = \sum\nolimits_{k = 1}^{N} {{\text{AMF}}_{i,k} /N}$$

where *i* represented the index of the targeted regions, *k* represented the index of baseline sample, *j* represented the index of dilution samples, *N* represented the count of the baseline samples.

### Subsampling data and model prediction

The model was built as our previous report [29]. Briefly, we developed a SVM classifier for PDAC plasma using tenfold cross-validation and support vector machine (SVM) with a cohort of data (54 healthy plasma and 63 PDAC plasma), which were sequenced on NovaSeq6000 in our last study [29], and employed the model to predict the data of collected plasma immediately in this study. To evaluate the robustness of the model performance of NovaSeq6000 and MGISEQ-2000, we observed the results of model prediction along with coverage of the targeted regions. We down-sampled the aligned data to 2 M, 1.5 M, 1.0 M, 0.5 M and 0.25 M using sambamba (0.8.1) [40] and repeated the process ten times. Then, these data were predicted with the model, respectively.

### Supplementary Information

**Additional file 1: Fig. 1.** The base quality score of the data of mTitan library. A, B, C, D. The base quality score of the data of mTitan library with 50% WGS library (**A**), 30% WGS library (**B**), 10% WGS library (**C**), and 0% WGS library (**D**). The base quality of R1 reads showed in left and that of R2 reads showed in right.

**Additional file 2: Fig. 2.** The fragment size of libraries. A, B. The libraries size distribution of mLib and iLib of synthetic cfDNA samples (**A**) and of clinical cfDNA samples (**B**). Colors represented library types.

**Additional file 3: Fig. 3.** The variation between the two sequencers. **A** The PC2’s rotation distribution. Dashed lines represented the cutoff of inter-sequencer’s highly variable regions (top 5% in PC2’s loadings). **B** The GC content of the inter-sequencer’s highly variable regions and random regions. The statistical analysis was performed by ‘wilcox.test’. **C** The correlation of methylation levels between MGISEQ-2000 and NovaSeq6000 upon differently methylated CpGs of synthetic cfDNA samples. We grouped the targeted CpG sites according to their methylation levels with a bin interval of 0.2. **D** The CHN methylation levels of synthetic cfDNA samples. The *y*-axis represented the methylation levels of CHN sites. The statistical analysis was performed by ‘wilcox.test’. **E** The correlation of methylation levels between MGISEQ-2000 and NovaSeq6000 upon differently methylated CpGs of clinical cfDNA samples. We grouped the targeted CpG sites according to their methylation levels with a bin interval of 0.2. **F** The CHN methylation levels of clinical cfDNA samples. The *y*-axis represented the methylation levels of CHN sites. The statistical analysis was performed by ‘wilcox.test’.

## Data Availability

The supporting data and code are now available in Github (https://github.com/sunjin1/MGISEQ2000_NovaSeq6000_BS_com). The raw sequence data reported in this paper have been deposited in the Genome Sequence Archive [41] in National Genomics Data Center [42], China National Center for Bioinformation/Beijing Institute of Genomics, Chinese Academy of Sciences (GSA-Human: HRA005151) that are publicly accessible at https://ngdc.cncb.ac.cn/gsa-human.

## Abbreviations

ctDNA: Circulating tumor DNA
cfDNA: Cell-free DNA
LOD: Limit of detection
NGS: Next-generation sequencing
SBS: Sequencing by synthesis
DNB: DNA NanoBalls
cPAS: Combined primer anchor synthesis
WGS: Whole genome sequencing
WES: Whole exome sequencing
meDNA: Standard fully methylated genomic DNA
BS: Bisulfite sequencing
AMF: Average methylation fraction
PCA: Principal component analysis
PDAC: Pancreatic ductal adenocarcinoma
QC: Quality control
gDNA: Genomic DNA
PCC: Pearson’s correlation coefficient
SVM: Support vector machine
AUC: Area under the curve
mLib: The sequencing library prepared by MGI official experimental kits
iLib: The sequencing library prepared by Illumina official experimental kits
