# Supplementary figures and images for: Cross-platform comparisons for targeted bisulfite sequencing of MGISEQ-2000 and NovaSeq6000
Source: Clin Epigenetics. 2023 Aug 14;15:130. doi: 10.1186/s13148-023-01543-4 (PMC10426093; doi:10.1186/s13148-023-01543-4)

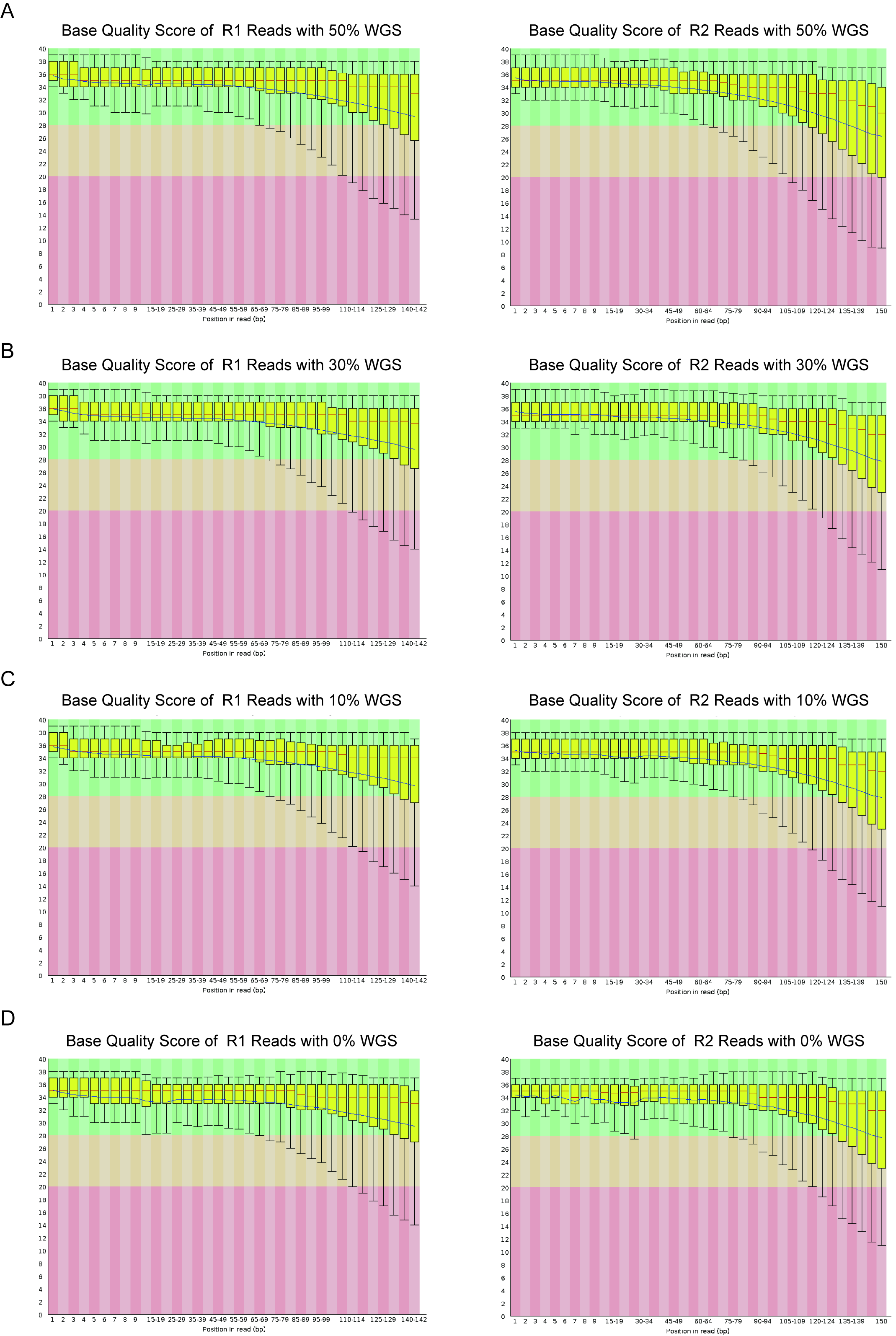

Supplement: Supplementary file 1 — Additional file 1: Fig. 1. The base quality score of the data of mTitan library. A, B, C, D. The base quality score of the data of mTitan library with 50% WGS library (A), 30% WGS library (B), 10% WGS library (C), and 0% WGS library (D). The base quality of R1 reads showed in left and that of R2 reads showed in right. [file 13148_2023_1543_MOESM1_ESM.tif]

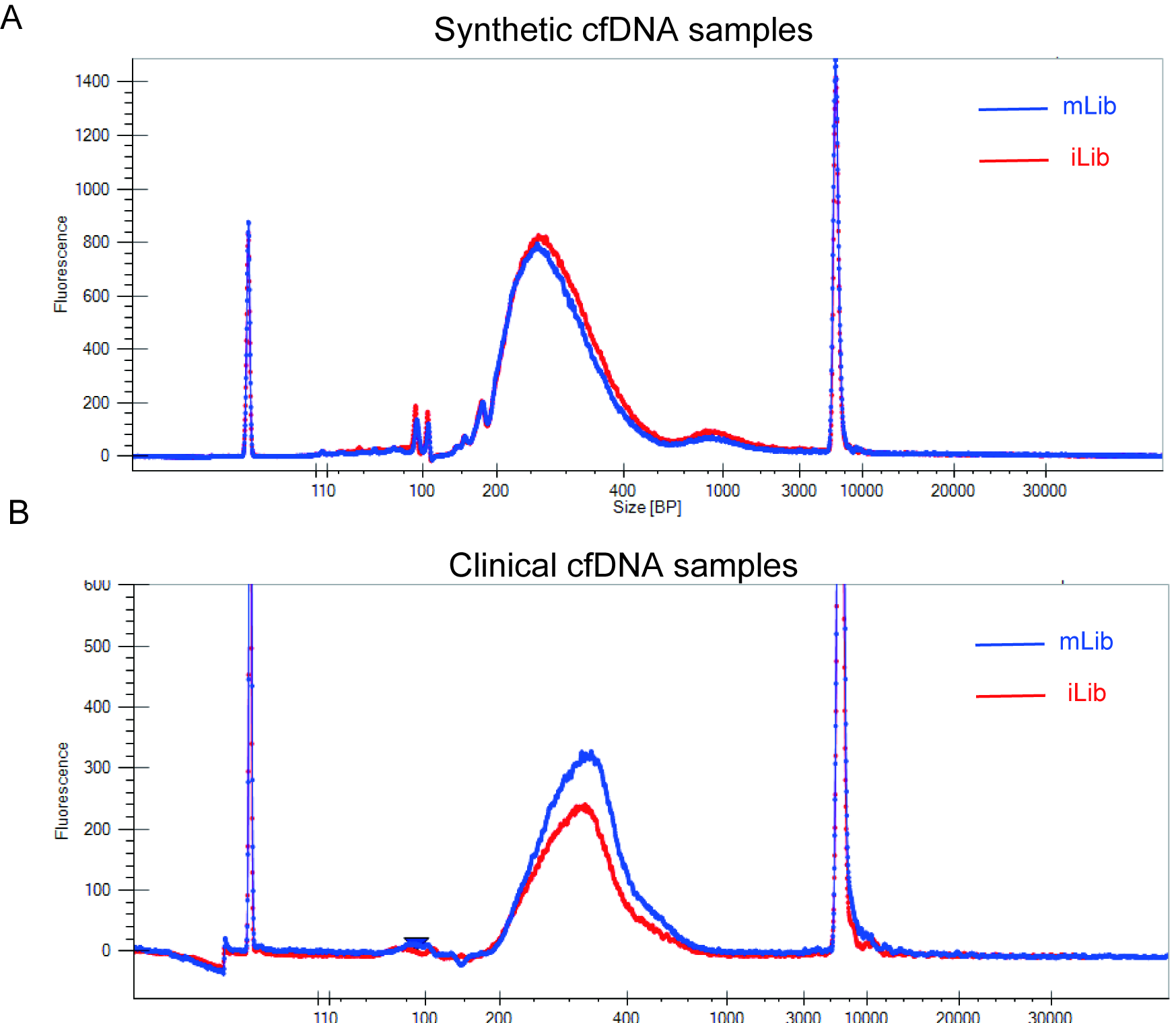

Supplement: Supplementary file 2 — Additional file 2: Fig. 2. The fragment size of libraries. A, B. The libraries size distribution of mLib and iLib of synthetic cfDNA samples (A) and of clinical cfDNA samples (B). Colors represented library types. [file 13148_2023_1543_MOESM2_ESM.tif]

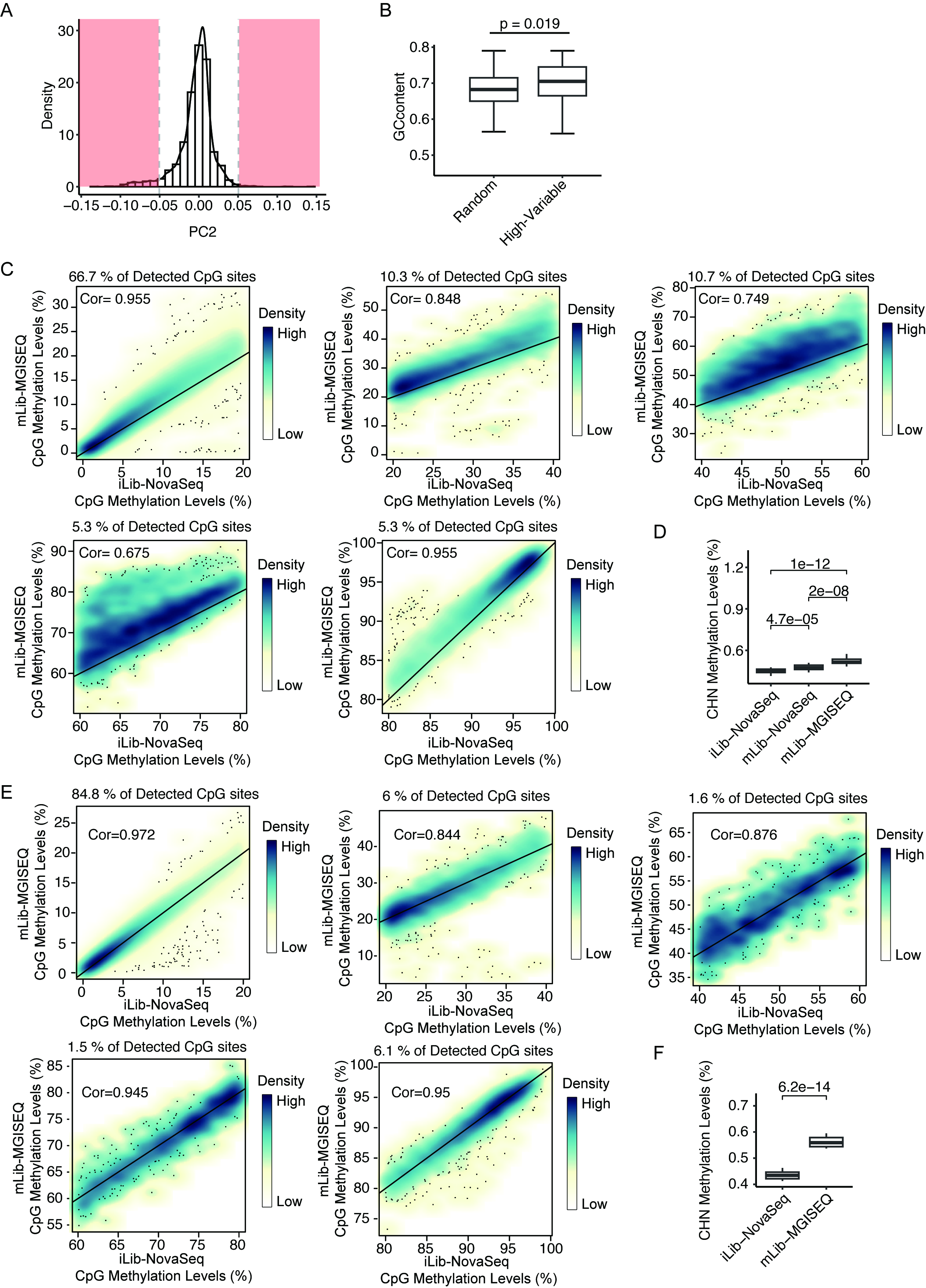

Supplement: Supplementary file 3 — Additional file 3: Fig. 3. The variation between the two sequencers. A The PC2’s rotation distribution. Dashed lines represented the cutoff of inter-sequencer’s highly variable regions (top 5% in PC2’s loadings). B The GC content of the inter-sequencer’s highly variable regions and random regions. The statistical analysis was performed by ‘wilcox.test’. C The correlation of methylation levels between MGISEQ-2000 and NovaSeq6000 upon differently methylated CpGs of synthetic cfDNA samples. We grouped the targeted CpG sites according to their methylation levels with a bin interval of 0.2. D The CHN methylation levels of synthetic cfDNA samples. The y-axis represented the methylation levels of CHN sites. The statistical analysis was performed by ‘wilcox.test’. E The correlation of methylation levels between MGISEQ-2000 and NovaSeq6000 upon differently methylated CpGs of clinical cfDNA samples. We grouped the targeted CpG sites according to their methylation levels with a bin interval of 0.2. F The CHN methylation levels of clinical cfDNA samples. The y-axis represented the methylation levels of CHN sites. The statistical analysis was performed by ‘wilcox.test’. [file 13148_2023_1543_MOESM3_ESM.tif]
